# Electrospinning of Bovine Split Hide Collagen and Collagen/Glycosaminoglycan for a Study of Stem Cell Adhesion and Proliferation on the Mats: Influence of Composition and Structural Morphology

**DOI:** 10.3390/jfb16060219

**Published:** 2025-06-12

**Authors:** Todorka G. Vladkova, Dilyana N. Gospodinova, Peter D. Dineff, Milena Keremidarska-Markova, Kamelia Hristova-Panusheva, Natalia Krasteva

**Affiliations:** 1 Department of Polymer Engineering, Faculty of Chemical Technologies, University of Chemical Technology and Metallurgy, 1756 Sofia, Bulgaria; 2 Department of Electrical Apparatus, Electro-Technical Faculty, Technical University of Sofia, 1797 Sofia, Bulgaria; dilianang@tu-sofia.bg (D.N.G.); dineff_pd@abv.bg (P.D.D.); 3 Institute of Biophysics and Biomedical Engineering, Bulgarian Academy of Sciences, BAS, 1113 Sofia, Bulgaria; m_keremidarska@uni-sofia.bg (M.K.-M.); kamelia.t.hristova@gmail.com (K.H.-P.); nataly@bio21.bas.bg (N.K.); 4 Department of Animal and Human Physiology, Faculty of Biology, Sofia University ‘St. Kliment Ohridski’, 1164 Sofia, Bulgaria

**Keywords:** bovine split hide collagen, glycosaminoglycan, electrospun fiber mats, benign solvents, human adipose-derived mesenchymal stem cells

## Abstract

Electrospun collagen-based fibrous mats are of increasing interest for cell culture, regenerative medicine, and tissue engineering. The focus of this investigation is on the assessment of the electrospinning ability of bovine split hide collagen (BSHC), the effect of glycosaminoglycan (GAG) incorporation on the mats’ structural morphology, and the impact on the adhesion and proliferation of human adipose-derived mesenchymal stem cells (hAD-MSCs). Electrospun mats were prepared using benign and fluoroalcohol solutions of BSHC and BSHC/GAGs under varied operation conditions. SEM observations and analysis were employed to characterize the structural morphology of the mats. Several parameters were used to evaluate the hAD-MSC behavior: cytotoxicity, cell morphology, cell number and spreading area, cytoskeleton, focal adhesion contacts, and cell proliferation. Electrospinning using benign solvents was impossible. However, fiber mats were successfully prepared from hexafluoropropanol (HFP) solutions. Different structural morphologies and fiber diameters of the electrospun mats were observed depending on the composition and concentration of the electrospinning solutions. Both BSHC and BSHC/GAG mats supported the in vitro adhesion, growth, and differentiation of hAD-MSCs, with some variations based on their composition and structural morphology. The absence of cytotoxicity and the good hAD-MSC adhesiveness make them promising substrates for cell adhesion, proliferation, and further stem cell differentiation.

## 1. Introduction

The rapid evolution of electrospinning technology has enabled the preparation of nanofiber mats with three-dimensional porous structures, which closely imitate the microstructure of the natural extracellular matrix (ECM). These materials have attracted increased attention as scaffolds for tissue engineering, drug delivery, wound dressing, periodontal regeneration, vascular reconstruction, etc. [1,2]

To date, a wide range of natural polymers (e.g., chitosan, glycosaminoglycans, and collagen) and synthetic polymers (e.g., polyglycolic acid (PGA), polylactic acid (PLA), and poly (lactic-co-glycolic acid) (PLGA)), as well as composite materials, have been successfully electrospun [3,4,5,6,7,8]. A critical factor in their effectiveness is their ability to closely mimic the topography and function of the natural extracellular matrix (ECM). Key parameters, such as the fiber diameter, diameter distribution, and alignment, significantly influence the properties of electrospun scaffolds [3]. Collagen, a major structural component of the ECM, is particularly advantageous due to its excellent biocompatibility, biodegradability, low immunogenicity, and ability to support cell and tissue growth. Electrospun collagen matrices are highly tunable, making them ideal for tailored tissue engineering [1,2,3,4,5]. Consequently, collagen has become a major component in the development of in vitro cell culture substrates, tissue engineering scaffolds, and drug delivery platforms. In these applications, the collagen used is derived from animal (bovine, porcine, equine, and chicken) and marine sources (jellyfish, sea cucumber, squids, starfish, sponges, crustaceans, and mussels), each offering specific properties. For instance, fish collagen demonstrates inherent antimicrobial activity, broadening its potential applications [6].

Compared to traditional collagen sponges and other porous materials, electrospun collagen and collagen composite matrices provide superior biomimicry, closely replicating the structural and mechanical properties of the native ECM [4,7,8,9]. The structural and functional properties of the electrospun collagen matrices depend on the electrospinning parameters as well as the collagen origin and type [10]. By optimizing the processing conditions, one can precisely control the fiber morphology to match specific tissue requirements [11].

Natural collagen fibrils serve as the primary structural element in load-bearing tissues such as tendons, ligaments, skin, cornea, and bone. A variety of electrospun collagen scaffolds can be engineered with a fiber diameter and porosity matching those of the native ECM, enhancing their regenerative potential [1,2,4]. Collagen is typically electrospun using fluoroalcohol solutions, but the degradation observed during electrospinning has led some researchers to question whether collagen electrospinning is merely an expensive way of producing gelatin [12]. Furthermore, concerns about potential toxicity from residual organic solvents in the electrospinning processes have driven interest in the development of alternative approaches using benign and aqueous-based systems. The first successful electrospinning of collagen nanofibers using phosphate-buffered solutions (a benign solvent system) was reported in 2009–2010 [13,14]. However, subsequent reports about electrospun collagen mats using similar mild solvents remain notably absent in the literature. The recent work of Visser et al. (2023) [15] has provided new insights through the enzymatic and spectroscopic analysis of electrospun rat tail collagen. Their study demonstrated that the triple-helical structure of collagen was disrupted when it was electrospun using acetic acid/ethanol mixtures at various temperatures, revealing that structural damage occurs independently of fluorinated solvents. This finding challenges the previous assumption that fluorinated solvents were solely responsible for collagen denaturation during electrospinning. The authors emphasize that the impact of electrospinning parameters on collagen’s biochemical properties must be carefully considered when fabricating ECM-mimicking fibrous constructs. While numerous electrospun porous mats fabricated from different collagen types and sources have been reported in the literature [1,2,4,6], no report was found about mats electrospun specifically from bovine split hide collagen (BSHC).

The development of composite collagen scaffolds that better mimic the natural extracellular matrix (ECM) has been carried out [7], including those biofunctionalized with molecules such as recombinant ICOS-Fc to enhance bone remodeling [16]. Among these, collagen/glycosaminoglycan mixtures are particularly promising for fabricating biomimetic scaffolds that provide not only mechanical support but also deliver biochemical signals to facilitate cell adhesion, proliferation, and differentiation [17,18]. However, challenges remain in ensuring these scaffolds accurately replicate the tissue-specific GAG expression profiles found in native human tissues, a critical consideration for their eventual commercialization [19]. The physical–chemical, mechanical, and biological properties of the collagen/GAG scaffolds can be precisely controlled by varying their composition (collagen origin and type; GAG type and amount) and the manufacturing conditions [20,21,22].

Collagen/GAG scaffolds have been extensively investigated as ECM analogues for regenerating a variety of tissues, including skin [23,24,25], peripheral nerves [26], muscles [27], cartilage [28], tendons [17,29], bone tissue [30,31], etc. Studies have also examined the behavior of different eukaryotic cells on collagen and collagen/GAG scaffolds, including the maturation of osteoblast cells [32,33]; osteoblast differentiation and matrix mineralization [34]; tendon cell recruitment, alignment, and metabolic activity [29]; human trabecular meshwork cell behavior [35]; different cell adhesion types [36], etc.

Stem cells possess two defining characteristics that distinguish them from other cell types: the ability to self-renew through prolonged proliferation and the capacity to differentiate into specialized cell lineages (e.g., myocytes, hematopoietic cells, and neurons). Among these, mesenchymal stem cells (MSCs) have emerged as particularly valuable for regenerative medicine due to their multipotent differentiation potential, immunomodulatory properties, and trophic factor secretion [37]. MSCs demonstrate remarkable plasticity in their biological responses, with their phenotype being highly sensitive to micro-environmental cues. This adaptability, combined with their multiline differentiation capacity (osteogenic, chondrogenic, adipogenic, etc.), makes them exceptionally versatile for therapeutic applications [38]. Adipose-derived mesenchymal stromal cells attract significant attention in both pre-clinical and clinical research due to their potential in treating a wide array of conditions, ranging from various autoimmune diseases such as inflammatory bowel disease, systemic sclerosis, type 1 diabetes, and rheumatoid arthritis, to neurological disorders including amyotrophic lateral sclerosis, ischemic stroke, and pinal cord injuries [39]. Notably, recent research has shown that MSCs exhibit significant antimicrobial activity through both indirect and direct mechanisms, partially mediated by the secretion of antimicrobial peptides and proteins [40]. These multifunctional properties position MSCs as promising candidates for addressing complex clinical challenges in regenerative medicine, antimicrobial therapy, and inflammation management.

Adipose-derived MSCs (AD-MSCs) were chosen for this study due to their high yield (100–500× more cells/gram than bone marrow), minimally invasive harvesting, and superior proliferation and differentiation potential [41,42]. Their strong adhesion to collagen-based scaffolds and robust immunomodulatory properties make them ideal for evaluating electrospun matrices. Compared to other MSC sources, AD-MSCs demonstrate better angiogenic potential, lower donor variability, and excellent cryopreservation stability, ensuring experimental reproducibility [43,44]. These advantages make AD-MSCs optimal candidates for tissue engineering applications requiring ECM interaction and regenerative capacity. Although electrospinning enables the fabrication of fibrous mats with properties closely resembling the natural ECM, which is ideal for seeding and growing different cell types, including MSCs [4], no previous studies have reported on the electrospinning of bovine split hide collagen (BSHC) or BSHC/glycosaminoglycan (BSHC/GAGs) composites.

This study aims to fabricate and characterize electrospun BSHC and BSHC/GAGs fibrous mats from benign and conventional solvents and to evaluate hAD-MSCs’ behavior on them. The structural morphology of the electrospun mats was optimized by systematically varying the concentration and composition of the BSHC and BSHC/GAGs solutions, applied voltage, flow rate, needle diameter, and tip-to-collector distance. Two distinct approaches, direct contact and indirect exposure to mats, were used to evaluate hAD-MSCs’ responses.

## 2. Experiment

### 2.1. Materials

Bovine split hide type I collagen, BSHC (Collapro Bovine Premium, Hulsh Protein Technologies, Oost Gelre, The Netherlands) was used in this investigation; Glutar aldehyde (GA, 50% water solution, Alfa Aesar, Karlsrue, Germany) was used as a cross-linking agent. Modifying agents were the glycosaminoglycans (GAGs) chondroitin sulfate (CS (Alfa Aesar, Heysham, UK) and hyaluronic acid, HA (Alfa Aesar, Heysham, UK), both as 10% water solutions.

The solvents 1,1,1,3,3,3-hexafluoro-2-propanol (HFP, 99.0%, Alfa Aesar, Heysham, UK) and ethyl alcohol (96.0%, p.a.), as well as a standard phosphate buffer (PBS, pH = 7.0 at 20 °C, CPA Chem, Bogomilovo, Bulgaria), were used in their delivered form.

All salts used for preparation of a concentrated phosphate buffer (PBS×20): NaCl, KCl, KH_2_PO_4_, and Na_2_HPO_4_·12H_2_O were p.a. Concentrated phosphate buffer, PBS×20, was prepared as follows: 80 g of NaCl, 2 g of KCl, 2 g of KH_2_PO_4_, and 28.8 g of Na_2_HPO_4_·12H_2_O was dissolved in 500 mL of distilled water, and pH was fixed at seven via addition of HCl.

### 2.2. Electrospinning Solutions

Based on reported advantages of the collagen electrospinning from benign solvents [13,14] and established methods using acetic acid/ethanol solutions [15], we prepared electrospinning solutions of BSHC in standard phosphate buffer (PBS), concentrated phosphate buffer (PBS20x), and mixed solvents: PBS/ethanol, PBS20x/ethanol, and acetic acid/ethanol. HFP electrospinning solutions of BSHC and BSHC/GAG were also prepared for this investigation.

#### 2.2.1. BSHC Solutions

- PBS and PBS20x solutions of BSHC

PBS and PBS20x solutions of BSHC with a step-varied concentration of 5–20 wt.% (step of 1 wt.%) were prepared via 3 h of intense stirring of BSHC preliminarily swelled in the corresponding solvent for 24 h.

- PBS/ethanol and PBS20x/ethanol solutions of BSHC

Solutions of BSHC with a step-varied concentration of 5–20 wt.% (step of 1 wt.%) in both PBS/ethanol (volume ratios, *v/v* 1:4; 1:2; and 1:1) and PBS×20/ethanol (volume ratio, *v/v* 1:1) were prepared via 3 h of intense stirring of BSHC preliminarily swelled in the corresponding mixed solvent for 24 h.

- Acetic acid/ethanol solutions of BSHC

Acetic acid/ethanol (*v*/*v*, 1:1) solutions of BSHC with concentrations of 6 wt.%, 8 wt.%, and 10 wt.% were prepared via 3 h of intense stirring of BSHC, preliminarily swelled in the mixture solvent for 24 h.

- HFP solutions BSHC

HFP solutions with step-varied concentration of 3–12 wt.% (step of 1 wt.%) were prepared by dissolving BSHC preliminarily swelled in HFP for 24 h.

#### 2.2.2. BSHC/GAGs Solutions

HFP solutions of BSHC/GAGs were prepared by premixing the corresponding GAG with BSHC, followed by adding HFP, 24 h of swelling at room conditions, and stirring for 3 h. The GAGs used in this investigation were hyaluronic acid (HA), chondroitin sulfate (CS), and combinations of them (HA/CS). Solutions with different amounts of GAGs were prepared as follows:

- Hyaluronic acid:
  BSHC/HA5 (5 wt.% HA), BSHC/HA10 (10 wt.%), BSHC/HA15 (15 wt.%).
- Chondroitin sulfate:
  BSHC/CS5 (5 wt.%), BSHC/CS10 (10 wt.%).
- Both hyaluronic acid and chondroitin sulfate:
  BSHC/HA5/CS5 (5 wt.% HA/ 5 wt.% CS);
  BSHC/HA10/CS5 (10 wt.% HA/ 5 wt. CS%);
  BSHC/HA10/CS10 (10 wt.% HA/10 wt.% CS).

### 2.3. Electrospinning

**Figure 1:**
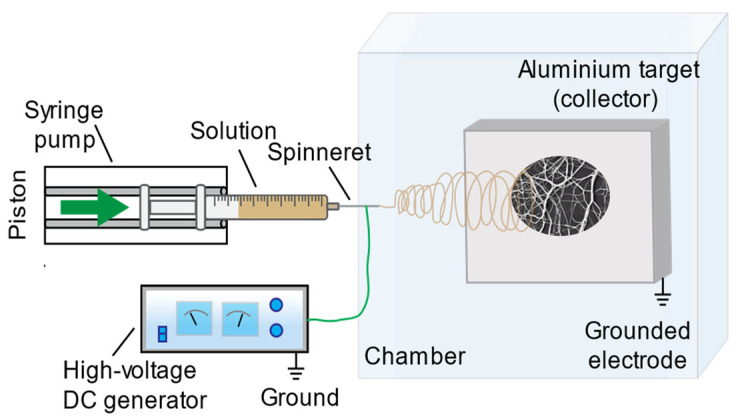
Schematic presentation of the electrospinning equipment employed for this investigation.

Numerous parameters are known to influence the electrospinning process, including solution properties (solvent type and solution concentration); process variables (flow rate, applied voltage, tip-to-collector distance, and needle diameter); as well as environmental characteristics, like temperature, humidity, etc. [1,2,4,45,46]. For this study, fibrous mats were electrospun under ambient laboratory conditions using the following operation conditions: electrospinning voltage, 10–30 kV; needle diameter, 0.4–0.9 mm; flow rate, 1–100 µLmin^−1^; and tip-to-collector distance, 10–30 cm for every solution. All experiments were conducted using a custom-built electrospinning apparatus (Figure 1), with parameters systematically varied for each solution formulation.

All electrospun samples were cross-linked via 24 h exposure in vapors of glutar aldehyde, which is one of the most commonly used cross-linking agents for collagen, among other others [47].

### 2.4. Scanning Electron Microscopy

The morphology of the electrospun BSHC and BSHC/GAG mats was observed using SEM (SEM/FIB Lyra I XMU, Tescan, Brno, Czech Republic). ImageJ, version 1.54g, NIH, USA, software was employed to accurately measure fiber diameter from the SEM images, analyzing 300 randomly selected areas.

### 2.5. Cell Experiments

- Cell culture conditions

Human adipose-derived mesenchymal stem cells (hAD-MSCs) were obtained from Lonza (Spain) at passage 2 and cultured in Dulbecco’s Modified Eagle Medium (DMEM) supplemented with 10% fetal bovine serum (FBS), 1% antibiotic/antimycotic solution (Sigma, Baden-Württemberg, Germany), and 2 mM.

L-glutamine (Roche Diagnostics, Mannheim, Germany) and 1 mM sodium pyruvate (Gibco BRL, Waltham, MA, USA) were used. The cells were incubated at 37 °C in a humidified atmosphere with 5% CO_2_. Media changes were performed twice a week. For experimental procedures, cells were detached using Trypsin-EDTA (Lonza, Verviers, Belgium), counted using a Neubauer haemocytometer, and seeded in 24-well plates. Depending on the experimental conditions, the cells were subsequently assayed according to the protocols outlined below.

- Indirect Cytotoxicity Evaluation

Cells were seeded onto plain cover glasses (CG) at a concentration of 2 × 10^4^ cells/mL and incubated in DMEM supplemented with 10% FBS for 24 h. After this incubation, the culture medium was replaced with medium pre-incubated for 4 days with different mats (NFs) to assess whether the mats release any toxic substances that could affect cell viability. After 2 and 24 h of incubation, the medium was removed, and the cells were stained with 0.001% fluorescein diacetate (FDA) (Sigma, Munich, Germany), dissolved in acetone, for 2 min. The cells were then washed several times with PBS, and representative images were captured using a fluorescent microscope (Zeiss, Axiovert 25, Oberkochen, Germany) equipped with a digital camera.

- Overall cell morphology, cell number, and spreading area

Human AD-MSCs were seeded onto studied mats at concentration of 2 × 10^4^ cells/mL and incubated in serum-free DMEM for 2 h. At the end of the incubation, the medium was removed, and the cells were processed for FDA staining, as described above. Representative images were captured using a fluorescent microscope. Plain (uncoated) glass coverslips (CG) and fibronectin (FN)-coated CG (20 μg·mL^−1^, Roche Diagnostics, Monza, Italy) served as negative and positive controls, respectively. Cell number and average cell spreading area were quantified using ImageJ software based on FDA-stained micrographs captured at 10x magnification. Data shown are representative of three separate experiments, and values are given as mean ± SD. Differences to control (FN-coated CG) with (*p*) < 0.05 and (*p*) < 0.01 were considered statistically insignificant and marked with one and two asterisks: * *p* < 0.05; ** *p* < 0.01.

- Immunostaining of actin cytoskeleton and focal adhesion contacts

Human AD-MSCs were seeded onto nanofibers, as previously described. After 2 h of incubation in serum-free medium, the cells were fixed with 3% paraformaldehyde, permeabilized with 0.5% Triton-X100 (*v*/*v*), and stained to visualize focal adhesion complexes and the actin cytoskeleton. Specifically, cells were stained with anti-vinculin antibody to visualize focal adhesion complexes or FITC-conjugated phalloidin to stain the actin cytoskeleton. After three washes with PBS, vinculin samples were incubated with a secondary antibody, followed by additional washing to remove unbound dye. Then, the cells were mounted and examined using a fluorescent microscope.

- Cell proliferation assay

Cells with concentration of 2 × 10^4^ cells/mL were seeded in 24-well plates containing collagen mats in DMEM supplemented with 10% FBS. At various time points, cells were transferred to a new plate, washed with PBS, and assessed for proliferation using the CCK-8 assay, according to the manufacturer’s instructions. The newly synthesized yellow formazan dye was quantified spectrophotometrically using a standard microplate reader (BioRad) at a wavelength of 450 nm. Values are expressed as means ± SEM (n = 3).

## 3. Results and Discussion

Electrospinning trials were performed for BSHC and BHSC/GAG using both benign solvents and HFP under a wide range of solution concentrations and electrospinning operation conditions. The structural morphology of the resulting electrospun BSHC and BSHC/GAGs mats was analyzed using SEM images, while the behavior of hAD-MSCs was evaluated using direct and indirect methods.

### 3.1. Electrospinning of BSHC from Benign Solvents

The electrospinning of collagen from fluoroalcohol solutions presents several challenges, including toxicity, high cost, and significant destruction of the triple-helical structure of the collagen [4]. Given previous reports on the electrospinning of collagen from benign solvents, such as phosphate buffers and their ethanol mixtures [13,14], as well as the successful use of acetic acid/ethanol for rat tail collagen [15], we initiated our study by exploring the electrospinning of BSHC from similar solvents. A wide range of operation conditions were tested for each BSHC solution, including voltages of 10 kV to 30 kV, flow rate from 1 µL/min to 100 µL/min, tip-to-collector distance of 10 cm to 30 cm, and needle diameters ranging from 0.4 mm to 0.9 mm.

Electrospinning of both PBS and PBS×20 solutions of BSHC across a broad concentration range (4–20 wt.% (step of 1 wt.%) was unsuccessful. Phase separation occurred in the mixed solvents, including PBS/ethanol (1:4, 1:2, 1:1, *v/v*) and PBSx20/ethanol (1:1, *v*/*v*), with ethanol forming the upper phase. As a result, electrospinning was not feasible under any tested conditions.

Similarly, attempts to electrospinning BSHC solutions prepared in acetic acid/ethanol (1:1, *v*/*v*) at concentrations of 4, 6, 8, 10, and 12 wt.% were unsuccessful under all operation conditions. Notably, the formation of Taylor cones was not observed, further indicating the unsuitability of these solvent systems for the electrospinning of BSHC. The extensive number of trials conducted across a wide range of solution concentrations and electrospinning conditions demonstrated that the fabrication of electrospun BSHC mats from benign solvents (phosphate buffers and their ethanol mixtures or acetic acid/ethanol) was not feasible. Therefore, subsequent experiments focused on the electrospinning of BSHC and BSHC/GAG mats using HFP solutions.

### 3.2. Electrospun BSHC Mats

The electrospinning of BSHC from HFP solutions (3–12 wt.%) exhibited spray formation at 3 wt.% and stopped the needle at 10 wt.% and 12 wt.% solutions under varied operation conditions, including voltage ranging from 10 kV to 30 kV, needle diameter of 0.6–0.9 mm, flow rates of 1–100 µL/min, and tip-to-collector distance of 10–30 cm.

Fibrous mats with different fiber diameters were successfully electrospun from 6–9 wt.% BSHC solutions in HFP at an applied voltage of 25 kV, flow rate of 50–60 µL/min, needle diameters of 0.7 and 0.9 mm, and tip-to-collector distance of 15 cm.

**Figure 2:**
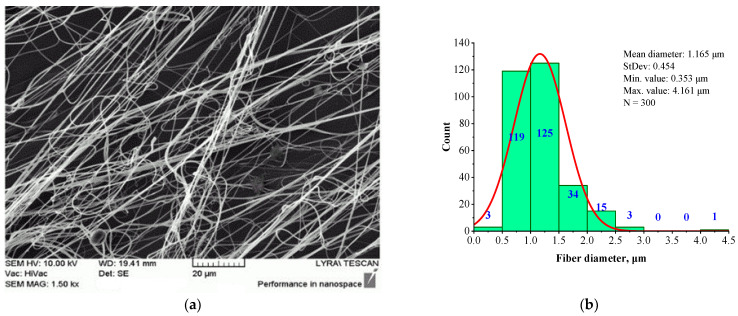
SEM image (**a**) and histogram (**b**) of BSHC fibrous mat electrospun from 6 wt.% HFP solution after cross-linking via 24 h of exposure in glutaraldehyde vapors.

Figure 2 presents the morphology (a) and fiber diameter distribution (b) of a fibrous mat electrospun from 6 wt.% BSHC solution in HFP, cross-linked via 24 h of exposure to glutaraldehyde vapors.

It is evident that the electrospun BSHC mat consists of defect-free fibers (Figure 2a) with micron and submicron diameters, and a relatively narrow fiber diameter distribution (Figure 2b). The mean diameter is 1.165 µm, with a standard deviation of 0.454 µm, a minimum value of 0.353 µm, and a maximum value of 3.003 µm.

**Figure 3:**
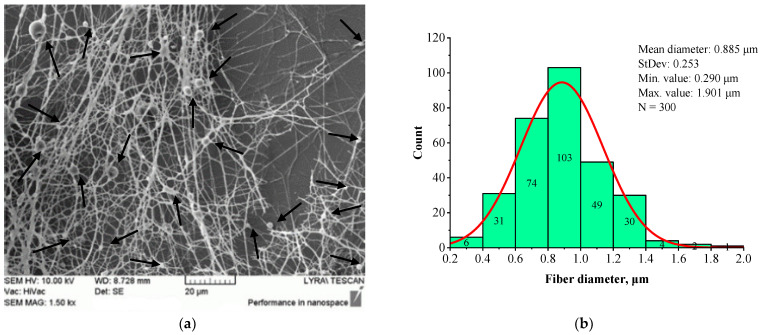
SEM image (**a**) and histogram (**b**) of BSHC mat electrospun from 9 wt.% HFP solution.

Submicron-diameter fibers are observed in the mats electrospun from 7 wt.% BSHC solutions (Figure 3b) and higher concentrations in HFP. However, these fibers exhibit defects in the form of balls, as shown in Figure 3a.

The SEM image (Figure 3a) reveals defects in the BSHC mat, whereas the corresponding histogram (Figure 3b) shows the formation of fibers with a mean diameter of 0.885 µm, a standard deviation of 0.253 µm, a minimum value of 0.290 µm, and a maximum value of 1.901 µm. Comparing this to the BSHC mat, electrospun from 6 wt.% HFP solution (Figure 2), it is evident that the electrospinning of BSHC from more concentrated HFP solutions (7 wt.% and above) results in finer fibers, but with noticeable defects. These trials demonstrate that electrospinning of BSHC from HFP solutions enables the fabrication of fibrous BSHC mats with fibers in the micron and submicron scale. However, to obtain defect-free fibers, the optimal concentration for electrospinning is between 5 wt.% and 6 wt.%.

### 3.3. Electrospun BSHC/GAG Mats

Glycosaminoglycans (GAGs) are a main component of the natural ECM of different tissues, playing an active role in regulating cell proliferation, differentiation, and tissue repair [48]. To create biomimetic scaffolds that better replicate the natural ECM, GAGs are often incorporated into porous collagen mats [22,34]. Collagen is most commonly combined with hyaluronic acid (HA) and chondroitin sulfate (CS), both of which are crucial for maintaining the ECM microenvironment and facilitating signaling molecules in living tissues [19,49,50]. Since the cells’ activity, including viability, proliferation, and distribution within the scaffold, depends on the composition and concentration of the GAGs [33,35], electrospun BSHC/GAG mats were fabricated for this study with varying concentrations of HA and CS, and combinations of both HA and CS.

The electrospinning conditions used to fabricate the BSHC/GAGs mats were optimized to produce defect-free fibers: 6 wt.% HFP solutions, an electrospinning voltage of 25 kV, a flow rate of 60 µL/min, a needle diameter of 0.7 mm, and a tip-to-collector distance of 15 cm. All samples were cross-linked via exposure to glutaraldehyde vapors for 24 h. The concentrations of HA, CS, and HA/CS were systematically varied, and the resulting mat samples were labeled as described in the Experiment section.

It was observed that when the total GAG concentration in the HFP solutions reached 15 wt.% or higher (samples BSHC/HA20, BSHC/HA10/CS5, and BSHC/HA10/CS10), electrospinning became impossible due to clogging of the needle. Fibrous mats were successfully electrospun from 6 wt.% HFP solutions containing lower GAG concentrations, including BSHC/HA5, BSHC/HA10, BSHC/CS5, BSHC/CS10, and BSHC/HA5/CS5.

**Figure 4:**
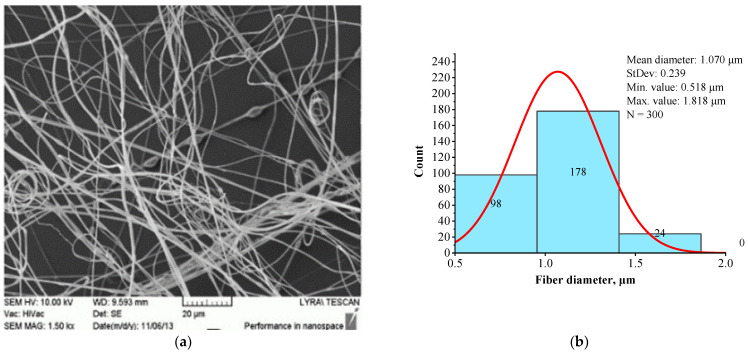
SEM image (**a**) and histogram (**b**) of BSHC/HA5 mat electrospun from 6 wt.% HFP solution of BSHC in presence of 5 wt.% HA.

The structural morphology (a) and fiber diameter distribution (b) of BSHC/HA5 fibrous mat, electrospun from 6 wt.% HFP solution containing 5 wt.% HA, are presented in Figure 4.

A comparison of Figure 3 and Figure 4 demonstrates that the presence of HA results in slightly finer, defect-free fibers with a relatively narrow fiber diameter distribution. The histogram in Figure 4b indicates that the average diameter of BSHC/HA5 fiber is 1.070 µm, with a standard deviation of 0.518 µm), whereas BSHC fibers have a slightly larger average diameter of 1.165 µm, with a standard deviation of 0.459 µm, as shown in Figure 3b.

**Figure 5:**
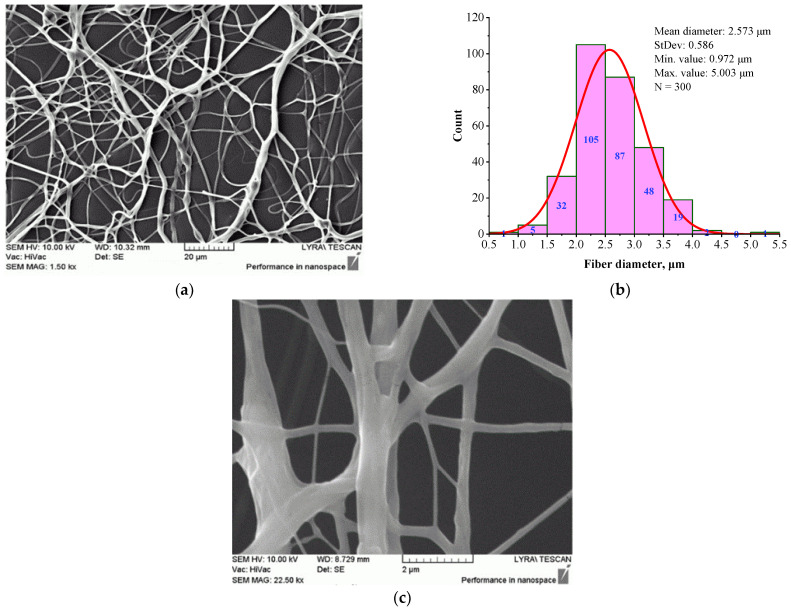
Morphology (**a**), (**c**) and histogram (**b**) of BSHC/CS5 mat, electrospun from 6 wt.% HFP solution of BSHC in presence of 5 wt.% CS, cross-linked in glutaraldehyde vapors for 24 h.

The presence of CS in the BSHC/CS5 solution significantly alters the structural morphology of the electrospun mat, as shown in Figure 5.

The fibers appear highly branched, as seen in Figure 5a, with greater clarity at high magnification, as shown in Figure 5c. The histogram in Figure 5b shows that their mean diameter is 2.573 µm (standard deviation of 0.972 µm), which is significantly larger than that of BSHC mat fibers (mean diameter of 1.165 µm, standard deviation of 0.459 µm, Figure 3b) and BSHC/HA5 mat fibers (mean diameter of 1.070 µm, standard deviation of 0.239 µm, Figure 4b). This indicates that the presence of CS in the BSHC electrospinning solution results in the formation of thicker fibers, whereas the presence of HA leads to the formation of finer fibers. The reason for the altered structural morphology of the BSHC/CS5 mat could be the possible hydrogen bonding of the CS to the collagen that has already been observed by other researchers [51]. The small amounts of the GAG used by us, both CS and HA (5 or 10 wt.% as 10% water solutions), make the detection of their hydrogen bonding to the collagen molecules difficult, and therefore, such experimental data are not presented here, the details can be found in the Supplementary Materials.

**Figure 6:**
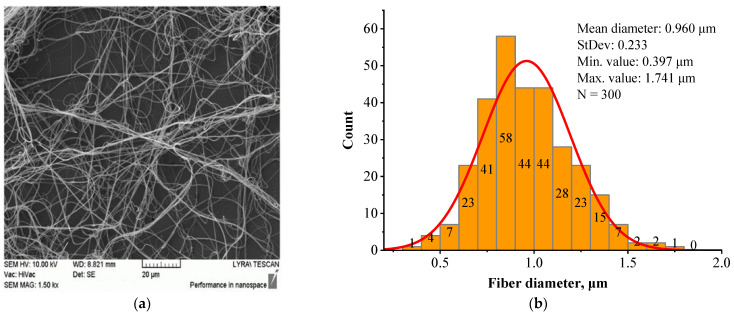
Morphology (**a**) and fiber diameter distribution (**b**) of the BSHC/HA5/CS5 mat electrospun from a 6 wt.% HFP solution of BSHC incorporating 5 wt.% HA and 5 wt.% CS cross-linked in glutaraldehyde vapors for 24 h.

Figure 6 presents the structural morphology (a) and fiber diameter distribution (b) of the BSHC/HA5/CS5 mat electrospun from the 6 wt.% HFP solution of BSHC containing both 5 wt.% HA and 5 wt.% CS.

In contrast to the highly branched fibers of the BSHC/CS5 mat containing CS (Figure 5a,c), the BSHC/HA5/CS5 mat, which incorporates both HA and CS, primarily consists of linear fibers. Their mean diameter of 0.960 µm (standard deviation: 0.233 µm, Figure 6b) is lower than that of the defect-free linear fibers of both BSHC (2.573 µm, standard deviation: 0.233 µm, Figure 2b), and BSHC/HA mats (1.070 µm, standard deviation: 0.518 µm, Figure 4b). Notably, the fibers in the BSHC/HA5/CS5 mat containing both HA and CS exhibit a high degree of entanglement.

It could be concluded that the structural morphology of the studied BSHC/GAGs fibrous mats, electrospun under identical operation conditions, is specific and dependent on their composition, as previously reported [48]. The fibers within these mats can be either linear or branched, exhibiting variations in mean diameter and diameter distribution, yet consistently falling within the micron and submicron scale. The presence of HA in BSHC/HA5 and BSHC/HA5/CS5 results in a lower mean diameter compared to the BSHC mat, which lacks HA. In contrast, the inclusion of CS in the BSHC/CS5 solution leads to the formation of mats with highly branched and relatively thick fibers. Notably, while the fibers in the BSHC/HA5/CS/5 mats are finer, they exhibit a greater degree of entanglement compared to other defect-free fibers.

### 3.4. Behavior of Human Adipose-Derived Mesenchymal Stem Cells (hAD-MSCs)

It is known that the behavior of human cells on porous collagen materials is influenced by their composition, structural morphology, and the collagen origin. GAGs are often incorporated to improve the biological activity of these materials. The type and concentration of GAGs significantly affect cell activity, including viability, proliferation, and spatial distribution within the scaffold [33,35,36,41]. In this study, we investigated electrospun fibrous mats based on BSHC and BSHC/GAGs with varying composition and structural morphology, which had not been previously studied. Table 1 demonstrates the selected electrospun mats for this investigation, with different compositions, structural morphologies, and fiber diameters.

Two distinct approaches, direct contact and indirect exposure, were employed to evaluate comprehensively the behavior of the hAD-MSCs [51]. The direct contact approach involves seeding cells directly onto the mats, providing insights into how their physical and bioactive properties influence cellular responses, including changes in cell attachment, membrane integrity, and morphology [52,53]. In contrast, the indirect exposure method assesses the potential release of toxic substances from the mats over time and allows for the evaluation of the impact of leached compounds on cell viability and morphology without direct physical interaction [54,55]. By combining these two approaches, a more comprehensive understanding can be gained regarding both the benefits and potential risks of the electrospun mats for biomedical applications.

#### 3.4.1. Indirect Method

The indirect cytotoxicity of the studied BSHC and BSHC/GAGs fibrous mats was evaluated using fluorescein diacetate (FDA) staining to observe cell viability and overall cell health after 2 and 24 h exposures to DMEM pre-incubated with the mats [56]. The fluorescence intensity directly correlates with the number of viable cells. Morphology changes such as cell rounding, shrinkage, or detachment were considered as indicators of a toxic effect.

**Figure 7:**
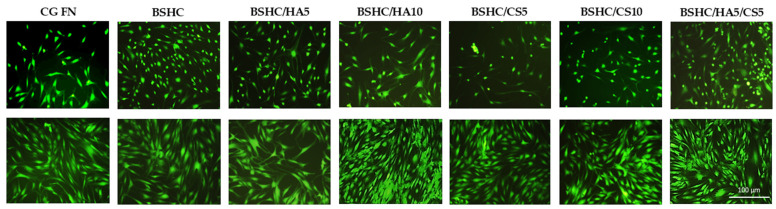
Indirect cytotoxicity toward hAD-MSCs cultured for 2 h (upper panel) and 24 h (lower panel) on positive control (CG-FN); BSHC mat without GAGs; and BSHC mats containing HA (BSHC/HA5 and BSHC/HA10), CS (BSHC/CS5 and BSHC/CS10), or both HA and CS (BSHC/HA5/CS5).

The indirect cytotoxicity of the studied mats toward the used test hAD-MSCs is presented in Figure 7.

The hAD-MSCs seeded on CG-FN (positive control) exhibit strong green fluorescence due to the enzymatic conversion of FDA to fluorescein, indicating healthy and viable cells. Some differences in cell viability were observed after 2 h of exposure to DMEM on all tested BSHC and BSHC/GAG mats compared to the CG-FN control (Figure 7, upper panel). After 24 h of exposure to DMEM, FDA fluorescence intensity increased in both control and all electrospun mats, suggesting cell proliferation and growth, with confluence being reached (Figure 7, lower panel). These findings indicate that any potential toxic substances leaching from the studied mats are negligible after 2 h and are over longer exposure periods (24 h and beyond). This highlights the biocompatibility of the electrospun pure BSHC mats, as well as those containing different amounts of HA, CS, or their combinations: BSHC, BSHC/HA5, BSHC/CS5, BSHC/CS10, BSHC/HA5/CS5, and BSHC/HA10/CS5.

#### 3.4.2. Direct Approach

In the direct approach, hAD-MSCs were seeded directly onto the electrospun mats with different compositions and structural morphologies, including BSHC, BSHC/HA5, BSHC/CS5, BSHC/CS10, BSHC/HA5/CS5, and BSHC/HA10/CS5. This allowed for a detailed assessment of hAD-MSCs’ viability, morphology, attachment, spreading area, and cytoskeletal organization.

- Overall cell morphology, cell number, and spreading area

**Figure 8:**
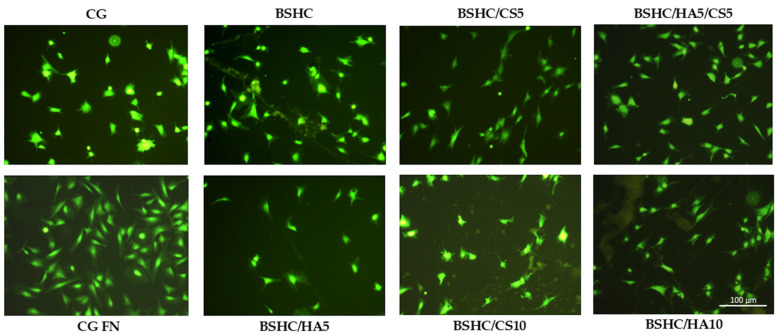
Fluorescence images of attached viable hAD-MSCs (stained with fluorescein diacetate, FDA) on negative control (CG), positive control (CG-FN), BSHC mat without GAGs, and BSHC mats containing HA (BSHC/HA5 and BSHC/HA10), CS (BSHC/CS5 and BSHC/CS10), or both HA and CS (BSHC/HA5/CS5).

The overall morphology of viable hAD-MSC cells after 2 h of attachment to the electrospun mats, BSHC, BSHC/HA5, BSHC/HA10, BSHC/CS5, BSHC/CS10, and BSHC/HA5/CS5, is shown in Figure 8. For comparison, a glass coverslip (CG) and fibronectin-coated glass coverslip (CG-FN) were used as negative and positive controls, respectively. Fibronectin, a highly adhesive extracellular matrix (ECM) protein known to promote robust cell adhesion and spreading [57,58,59], was chosen as a positive control.

Figure 8 illustrates that hAD-MSCs cultured on CG-FN (positive control) exhibit strong adhesion and spreading, displaying a characteristic fibroblast-like, spindle-shaped, and elongated morphology [60]. These cells’ features, a centrally located nucleus, thin cytoplasmic extensions, prominent filopodia (finger-like projections), and lamella podia (sheet-like extensions) at their edges, are indicative of strong adhesion and active cytoskeletal organization [60]. In contrast, hAD-MSCs on CG (negative control) appear more compact, shrunken, less spread, and display irregular protrusions. As a result, the hAD-MSCs exhibit weak adhesion, failing to develop leading and trailing edges typically observed in the FN-coated positive control, CG-FN. This weaker attachment also results in the absence of directed projections such as lamella podia and filopodia, which are hallmarks of cells interacting with more adhesive surfaces [61].

Favorable spreading of hAD-MSCs on the studied electrospun BSHC and BSHC/GAGs mats was expected, as their major component, collagen fibers, is well known to promote cell adhesion and growth. Moreover, the presence of HA, CS, or their combination was expected to further enhance cell attachment and proliferation, contributing to a more supportive microenvironment [62,63,64,65].

Significant morphological differences were observed among hAD-MSCs seeded on the studied electrospun mats, BSHC mat, and BSHC/GAGs mats (BSHC/HA5, BHSC/HA10, BSHC/CS5, BSHC/CS10, and BSHC/HA5/CS5) and compared to the negative (CG) and positive (CG-FN) controls (Figure 8). Cells on the BSHC mat, which lacks GAGs, exhibited the most spreading. In contrast, on the BSHC/CS10 mat, some cells appeared shrunken, while others displayed a stellate-like morphology, indicating poor adhesion. Notably, the BSHC mat consists of micron-diameter linear fibers, whereas the second one, BSHC/CS10 containing CS, consists of micron-diameter, highly branched fibers. Our study confirms that the fiber alignment has a significant effect on cellular behavior, inducing cell adhesion and proliferation [66].

Although fewer in number, hAD-MSCs on the electrospun BSHC/HA5 and BSHC/HA10 mats, both composed of micron-diameter linear fibers, also exhibited an elongated morphology, suggesting that HA may provide some degree of contact guidance influencing their morphology, as presented in [67].

**Figure 9:**
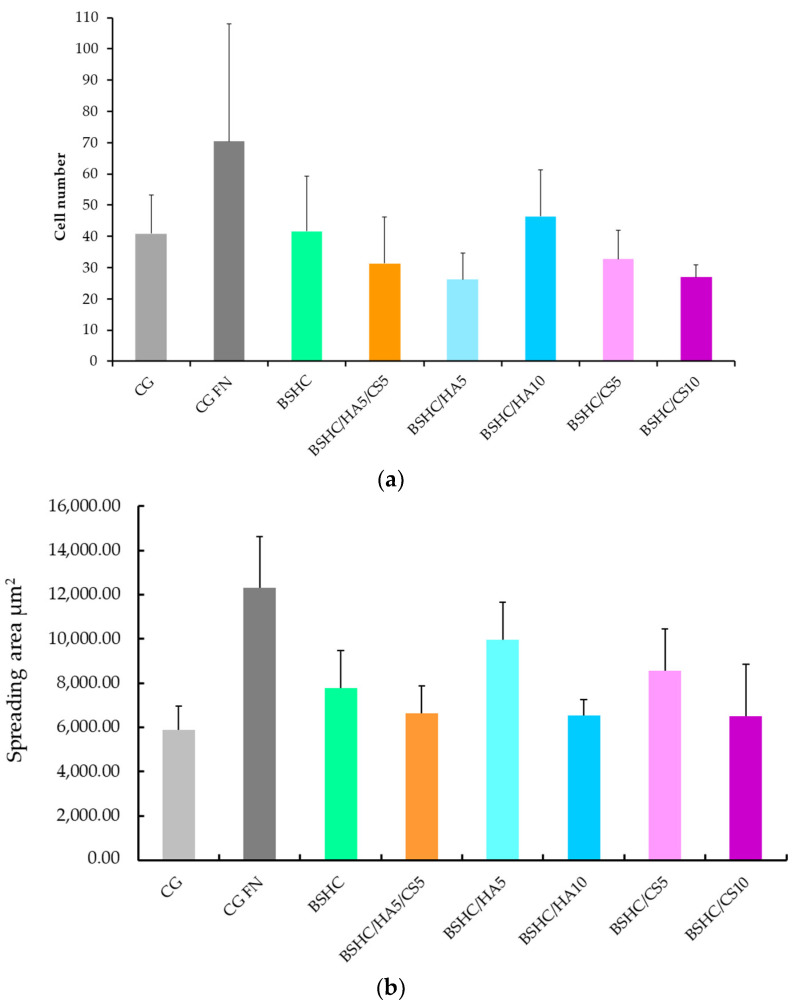
Number (**a**) and spreading area (**b**) of attached hAD-MSCs on negative control (CG), positive control (CG-FN); BSHC mat without GAGs; and BSHC mats containing HA (BSHC/HA5 and BSHC/HA10), CS (BSHC/CS5 and BSHC/CS10), or both HA and CS (BSHC/HA5/CS5).

The results for the number of attached cells (a) and the spreading area (b) are shown in Figure 9.

As expected, the number of adhered hAD-MSCs on the negative control, CG (Figure 9a, light gray column), is significantly lower than on the positive control, CG-FN (Figure 9b, the dark gray column). The number of adhered hAD-MSCs on the studied electrospun mats (Figure 9a, the green, orange, blue and rose columns) is also lower than on the positive control, CG-FN (Figure 9a, dark gray column) suggesting that the adhesiveness of BSHC and BSHC/GAGs mats for hAD-MSCs may be lower that of fibronectin.

The number of adhered hAD-MSCs varies among the studied electrospun mats, both those without GAGs (Figure 9a) and those containing GAGs (Figure 9a, the green, orange, blue, and rose columns). These differences likely reflect the influence of the variations in composition and structural morphology, which is a known effect [25].

The number of attached hAD-MSCs on the BSHC/HA10 mat with a lower fiber diameter is higher than that on the BSHC/HA5 mat, for which the fiber diameter is higher. The number of attached hAD-MSCs on the BSHC/HA10 mat with a lower branched fiber diameter is also higher than that on the BSHC/HA5 mat, for which the branched fiber diameter is higher (see Table 1). This confirms the known influence of the fiber’s diameter on cell adhesion [67].

Among the tested mats, BSHC/HA10 (dark blue column) exhibited the highest number of attached hAD-MSCs, followed by the BSHC mat without GAGs (the green column). Both are composed of non-branched fibers with micron-scale diameters. In contrast, BSHC/HA5 (light blue column) shows the lowest number of attached cells. The difference in cell attachment between BSHC, BSHCHA5, and BSHC/HA10 mats may be attributed to variations in the fiber thickness and the fiber diameter distribution, despite the three having fiber diameters within the micron-scale range (see Figure 2 and Figure 4 and Table 1). This is one more confirmation of the known fiber diameter’s influence on the cells’ attachment to electrospun collagen matrices [67].

Figure 9b reveals an intriguing observation: despite having the lowest number of attached cells, hAD-MSCs on BSHC/HA5 (light blue column) exhibit the largest spreading area, approaching that of cells on fibronectin-coated glass (Figure 9, dark gray column). This suggests that while certain electrospun mats may not support optimal initial cell attachment, they may still promote significant cell spreading when adhesion is weaker. These findings highlight a potential compromise between cell attachment and spreading on fibrous surfaces, emphasizing that the composition and structural morphology of the mats play distinct roles in regulating hAD-MSCs’ behavior [67].

- Organization of actin cytoskeleton

By visualizing the actin distribution, the effects of mat fibers on cell adhesion, spreading, and cytoskeletal dynamics can be assessed, providing valuable insight into how hAD-MSCs attach to both the mat fibers and the control surfaces. To further investigate cytoskeletal integrity, immunostaining was performed for the cytoskeletal protein F-actin. The arrangement of actin filaments plays a crucial role in determining cell morphology, influencing whether cells appear round, spread, or elongated. Disruptions of actin filament organization can serve as an indicator for cellular stress or cytotoxicity [67,68]. Specifically, fragmented or disorganized actin structures may suggest that mat fibers interfere with normal cell function or trigger apoptotic changes [65]. Additionally, actin filaments are essential for focal adhesion contact formation, where cells anchor to the extracellular matrix and surfaces [66,67].

**Figure 10:**
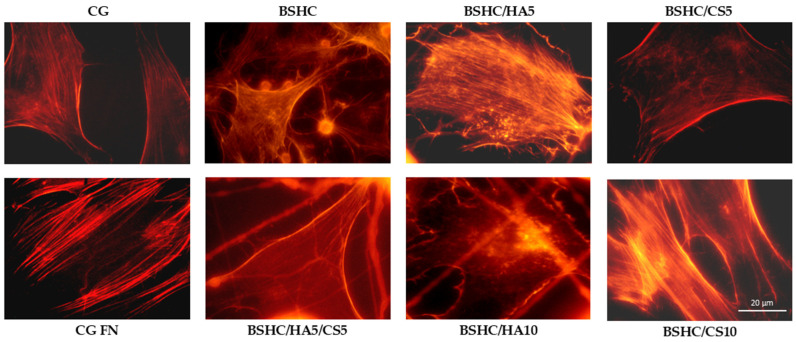
Organization of actin cytoskeleton in hAD-MSCs cultured in serum-free medium for 2 h on negative control (CG); positive control (CG-FN); BSHC mat without GAGs; and BSHC mats containing HA (BSHC/HA5 and BSHC/HA10), CS (BSHC/CS5 and BSHC/CS10), or both HA and CS (BSHC/HA5/CS5).

Figure 10 presents the organization of the actin cytoskeleton in hAD-MSCs cultured on control surfaces and the studied electrospun BSHC and BSHC/GAGs mats.

Actin is well-expressed across all studied electrospun mats, both those without GAG (BSHC) and those containing GAGs (HA, BSHC/HA5, and BSHC/HA10; CS, BSHC/CS5, and BSHC/CS10 or a combination of both HA and CS, BSHC/HA5/CS5). This indicates that these mats provide a suitable substrate for cell attachment and growth. Actin filaments are observed not only at the cell periphery but also throughout the cell body, supporting cell spreading and maintaining structural integrity. This suggests that the studied mats effectively promote cytoskeletal organization and facilitate cellular interactions, reinforcing their potential as biocompatible materials for tissue engineering applications.

- Vinculin Expression and Focal Adhesion Formation on Electrospun Fibrous Mats

Focal adhesion contacts are particularly important in the context of biomaterials, as they reflect how well cells interact with a surface. Well-formed focal adhesion contacts indicate strong cell-substrate adhesion and proper cytoskeletal organization, which are key markers of biocompatibility. Controversially, poorly formed or absent focal adhesion contacts may suggest weak adhesion, cellular stress, or cytotoxic effects, often resulting from suboptimal surface properties or material toxicity [66,69].

**Figure 11:**
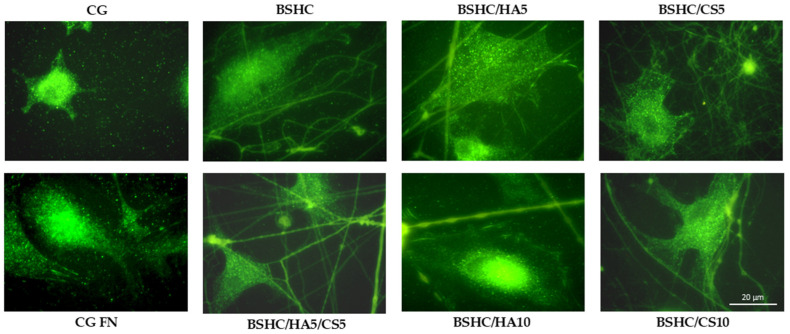
Organization of focal adhesion contacts in hAD-MSCs cultured for 2 h in a serum-free medium on negative control (CG); positive control (CG-FN); BSHC mat without GAGs; and BSHC mats containing HA (BSHC/HA5 and BSHC/HA10), CS (BSHC/CS5 and BSHC/CS10), or both HA and CS (BSHC/HA5/CS5).

To assess these interactions, immunostaining for vinculin, a key structural and regulatory protein in focal adhesion complexes, was performed. Vinculin plays a pivotal role in the cell attachment to the extracellular matrix (ECM) or synthetic substrates by linking actin filaments to adhesion plaque proteins, such as talin and paxillin. This molecular bridging is essential for transmitting mechanical forces; regulating cell signaling pathways; and influencing cell behavior, spreading, migration, and differentiation [66,68]. Figure 11 depicts the distribution and morphology of focal adhesion contacts in hAD-MSCs cultured on negative and positive controls, CG and CG-FN, respectively, and on electrospun BSHC and BSHC/GAGs mats.

Immunostaining analysis revealed that vinculin is well expressed in the hAD-MSCs adhered to all studied fibrous mats (BSHC and BSHC/GAG), confirming the active formation of focal adhesion complexes. Notably, well-defined focal adhesions were predominantly observed on BSHC, BSHC/HA5, and BSHC/HA10, composed of linear micron-diameter fibers. This suggests that electrospun mats with linear fiber architectures provide a more favorable substrate for focal adhesion assembly, probably due to their optimized structural and compositional properties. The presence of vinculin-rich focal adhesions on these substrates (BSHC, BSHC/HA5, and BSHC/HA10) underscores their ability to support stable cell adhesion and proper cytoskeletal organization, both critical for cell function and survival.

The observed variation in focal adhesion formation in the hAD-MSCs across the studied electrospun mats highlights the critical influence of scaffold composition and morphology on cell behavior. While BSHC, BSHC/HA5, and BSHC/HA10 demonstrated superior focal adhesion formation, other samples may require further optimization to improve their adhesive performance. These findings emphasize the importance of tailoring scaffold design to promote robust cell–material interaction.

- Proliferation of hAD-MSCs on Electrospun Fibrous Mats

The evaluation of cell proliferation is a critical aspect of assessing the biocompatibility and cytotoxicity of biomaterials, as it reveals their ability to support long-term cell viability and function. Proliferation studies not only indicate whether a material provides a favorable environment for cell growth but also detect the leaching of harmful substances that could impair cellular health over time [51]. In this study, we examined the proliferation of hAD-MSCs on electrospun fibrous mats to determine whether these scaffolds support the normal division and expansion or can induce inhibitory or cytotoxic effects that could compromise their suitability for biomedical applications [70].

**Figure 12:**
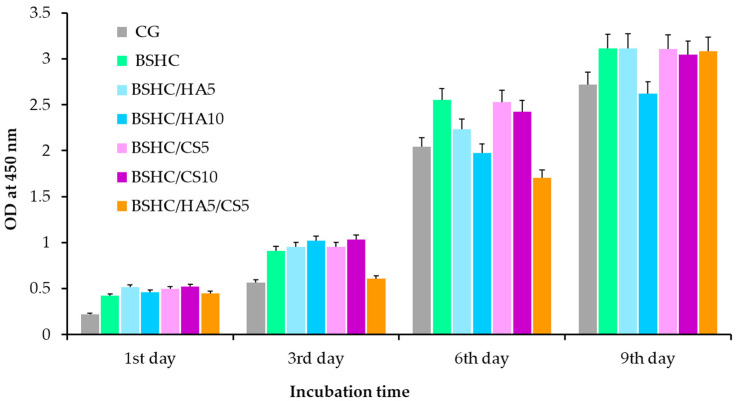
Proliferation of hAD-MSCs cultured for 9 days on control glass (CG); BSHC mat without GAGs; and BSHC mats containing HA (BSHC/HA5 and BSHC/HA10), CS (BSHC/CS5 and BSHC/CS10), or both HA and CS (BSHC/HA5/CS5).

The proliferation kinetics of hAD-MSCs cultured on the electrospun fibrous mats for up to 9 days are presented in Figure 12.

Figure 12 demonstrates that all electrospun mats, including the base BSHC mat and GAG-functionalized variants (BSHC/HA5, BSHC/HA10, BSHC/CS5, BSHC/CS10, and BSHC/HA5/CS5), significantly stimulate the growth of hAD-MSCs. The number of hAD-MSCs increases approximately 6-fold at the 9th day compared to the 1st day, indicating robust cell proliferation across all scaffolds. Notably, proliferation rates on BSHC and BSHC/GAGs mats are higher than those observed on the control glass, CG (Figure 12, colored vs. gray columns), suggesting these electrospun mats better replicate the native ECM by providing optimal structural morphology and biochemical cues to support cell attachment, spreading, and expansion.

While statistically insignificant differences were observed among mats at individual time points (days 1, 3, 6, and 9), minor variations may arise from the different composition and fiber architecture. For example, some mats may promote faster initial cell attachment, while others may support sustained proliferation longer. Despite these variations, the overall trend indicates that all tested mats support long-term cell growth without cytotoxicity and position them as promising candidates for applications requiring stable cell expansion and tissue maturation.

The results from the hAD-MSCs behavior study demonstrate that electrospun BSHC and BSHC/GAG mats support hAD-MSCs adhesion, spreading, and proliferation without cytotoxicity. Our key findings reveal the structural influence, compositional effects, adhesion-proliferation dynamics, and biocompatibility of the studied electrospun BSHC and BSHC/GAGs fibrous mats and confirm what is known from various scientific reports [25,51,67,71,72,73,74,75,76,77,78].

The findings can be summarized as follows:

1. The structural influence: Optimal cell spreading occurs on linear micro/submicron fiber mats (BSHC, BSHC/HA5, and BSHC/HA10); the fiber architecture (linear vs. branched) significantly impacts focal adhesion formation; mats with linear fibers provide superior mechanical support for cell attachment.
2. Compositional effects: HA-containing mats (BSHC/HA5) show exceptional cell spreading, rivaling fibronectin controls; GAG incorporation influences initial adhesion but not long-term proliferation; all compositions maintain cytoskeletal integrity and actin expression.
3. Adhesion–Proliferation Dynamics: While adhesion rates vary among mats, all support long-term proliferation (6-fold increase by day 9)*,* an inverse relationship exists between the adhesion density and spreading area, and the composition/structure differentially regulates attachment versus spreading behavior.
4. Biocompatibility: Rapid confluence was achieved within 24 h across all mats; no indirect cytotoxicity was observed, and all mats supported sustained growth comparable to controls.

These findings position BSHC-based electrospun mats as promising scaffolds for tissue engineering and regenerative medicine. The linear fiber mats (particularly HA-containing variants) offer an optimal balance of structural and biochemical cues for stem cell maintenance. While adhesion rates may vary, all compositions demonstrate excellent biocompatibility and proliferation support, making them suitable substrates for further differentiation studies.

## 4. Conclusions

The electrospinning of BSHC from benign solvents, such as phosphate buffers and their mixtures with ethanol, as well as acetic acid/ethanol solutions, was found to be unfeasible for concentrations ranging from 5 wt.% to 25 wt.%. This held true across a wide range of operational conditions, including voltages between 15 and 30 kV, flow rates from 1 to 100 µL/min, nozzle diameters between 0.5 and 0.9 mm, and tip-to-collector distances from 15 to 18 cm.

New electrospun BSHC and BSHC/GAG mats with distinct structural morphologies (micron- and submicron-diameter linear and branched fibers) were successfully prepared from HFP solutions. These mats were created both in the absence of GAGs and in the presence of hyaluronic acid (HA), chondroitin sulfate (CS), or a combination of both HA and CS.

The influence of different compositions and structural morphologies of the electrospun BSHC and BSHC/GAG mats was evaluated using both direct and indirect methods on human adipose-derived mesenchymal stem cells (hAD-MSCs). Parameters such as cell adhesion, spreading, focal adhesion contact formation, and proliferation were assessed. The electrospun BSHC and BSHC/GAG mats were found to support the long-term viability and proliferation of hAD-MSCs without inducing cytotoxicity. Additionally, the mats exhibited excellent hAD-MSC adhesiveness.

The behavior of hAD-MSCs on the BSHC and BSHC/GAG electrospun mats indicated that these mats do not induce indirect cytotoxicity and support hAD-MSCs’ adhesion and proliferation effectively. Therefore, BSHC and BSHC/GAG mats are promising candidates as substrates for cell adhesion and proliferation, and potentially for stem cell differentiation applications.

## Figures and Tables

**Table 1:** Structural morphology and composition of mats used to study hAD-MSCs’ behavior: those not containing GAGs (BHSC), those containing 5 wt.% or 10 wt.% HA (BSHC/HA5 and BSHC/HA10, respectively), those containing 5 wt.% or 10 wt.% CS (BSHC/CS5 and BSHC/CS10, respectively), and those containing 5 wt.% HA and 5 wt.% CS simultaneously (BSHC/HA5/CS5).

Sample	Structure	Mean Fiber Diameter (µm)	Min Diameter (µm)	Max Diameter (µm)
BHSC	Linear	1.165 ± 0.454	0.353	3.003
BSHC/HA5	Linear	1.070 ± 0.518	0.518	1.818
BSHC/HA10	Linear	0.832 ± 0.270	0.129	1.707
BSHC/CS5	Branched	2.573 ± 0.586	0.972	5.003
BSHC/CS10	Branched	2.196 ±0.838	0.705	5.193
BSHC/HA5/CS5	High degree of entanglement	0.960 ± 0.233	0.397	1.741

## Data Availability

The original contributions presented in this study are included in the article. Further inquiries can be directed to the corresponding author.

## Supplementary Materials

The following supporting information can be downloaded at: https://www.mdpi.com/article/10.3390/jfb16060219/s1.

## Abbreviations

The following abbreviations are used in this manuscript:

hAD-MSCs	Human adipose-derived mesenchymal stem cells.
BSHC	Bovine split hide collagen.
DMEM	Dulbecco’s Modified Eagle Medium.
MSCs	Mesenchymal stem cells.
ECM	Extracellular matrix.
GAG	Glycosaminoglycan.
SEM	Scanning electron microscopy.
HFP	Hexafluoropropanol.
BPS	Standard phosphate buffer.
CG-FN	Cover glass fibronectin coated.
GA	Glutar aldehyde.
CS	Chondroitin sulfate.
CG	Cover glass.
