# Supplementary material for: Electrospinning of Bovine Split Hide Collagen and Collagen/Glycosaminoglycan for a Study of Stem Cell Adhesion and Proliferation on the Mats: Influence of Composition and Structural Morphology
Source: J Funct Biomater. 2025 Jun 12;16(6):219. doi: 10.3390/jfb16060219 (PMC12194247; doi:10.3390/jfb16060219)
Supplement: Supplementary file 1 [file jfb-16-00219-s001.zip › jfb-3604247-supplementary.pdf]

Journal of
Functional Biomaterials

Article

Electrospinning of Bovine Split Hides Collagen and Collagen/Glycosaminoglycan for a Study of Stem Cell Adhesion and Proliferation on the mats: Influence of Composition and Structural Morphology

Todorka G. Vladkova [1,*], Dilyana N. Gospodinova [2], Peter D. Dineff [2], Milena Keremidarska-Marova [3], Kamelia Hristova-Panusheva [3] and Nataliya Krasteva [3]

 https://doi.org/10.3390/xxxxx

limitations

The results are specific to this collagen and care should be taken before extrapolating to other collagen types.

The GAG limit was set by the practical challenge of blockage free electrospinning at higher GAG content.

Amide band localization and HA/Cs analysis is a future possibility using spectroscopy, though the low GAG could compromise identification.

Based on the protocol used, however, incorporation is highly likely. Future surface characterisation coupled with cell outcome will facilitate understanding of mechanisms, not undertaken here.

Cell study is limited to the scaffold surfaces here, but scaffold profiling can be considered in future using confocal microscopy.

Though only live/dead profiling of cells was undertaken here, more robust, less photo bleachable dyes in future will enable minor cell changes to be examined.
